# Technical Aspects of Addressing Lower Buttock Ptosis and Contouring: The Thong Lift Technique

**DOI:** 10.1093/asjof/ojaf100

**Published:** 2025-08-08

**Authors:** Kishan S Shah, Cameron J Sabet, Paul E Chasan

## Abstract

**Background:**

Recent advancements in buttock aesthetics have largely focused on fat transfer, implants, and belt line lifts, all of which overlook the specific needs of the lower pole of the buttock, especially the inferomedial region. These areas are characterized by ptosis, exaggerated infragluteal creasing, and an L-shaped/boxy appearance, requiring targeted intervention.

**Objectives:**

The authors of this study aim to investigate the utilization of an infragluteal thigh lift, termed the thong lift (TL), to correct isolated lower buttock ptosis and L-shaped/boxy buttock deformity.

**Methods:**

A retrospective chart review and patient survey was conducted for consecutive patients who underwent the TL procedure at a single surgical center between January 2017 and April 2024. Clinical data collected included patient demographics, indications, surgical history, and postoperative outcomes. Patients were also administered a standardized satisfaction survey following the procedure to rate outcomes.

**Results:**

In total, 26 consecutive female patients (mean age: 48.5 years, range, 27-69 years) underwent the TL procedure during the study period. The average follow-up was 48.7 months. Patient satisfaction was high, with contour and scar quality rated as “good” to “excellent,” on average. All patients (100%) reported improved aesthetic appearance and satisfaction with their outcomes. Only minor complications were recorded (15.4%), all of which were resolved.

**Conclusions:**

Based on the results of this study, the TL is a promising technique that can be utilized to correct lower buttock ptosis and contour irregularities overlooked by traditional techniques, offering a targeted solution with minimal complications and high patient satisfaction.

**Level of Evidence: 4 (Therapeutic):**

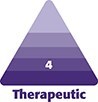

Buttock surgery is a staple of modern-day plastic surgery, with over 29,816 buttock augmentations (implants and fat transfer) and over 12,097 buttock lifts performed in 2023, according to the American Society for Aesthetic Plastic Surgery annual report.^1^ Although the traditional approaches to buttock lifting surgery focus on optimizing buttock volume and lateral contours, lower pole contouring often times remains overlooked.^2-4^ Procedures like fat transfer, buttock implants, and traditional buttock lifts add overall volume but lack the precise control needed to treat specific lower buttock conditions: isolated ptosis, excessive infragluteal creasing, and the L-shaped or “boxy” deformity that is often seen in females.^5,6^ These conditions can compromise and diminish the smooth contours patients are often seeking, and they remain difficult to correct with current treatments. Additionally, many patients undergo lower pole or “banana-fold” liposuction in an attempt to enhance the shape and contour of the lower buttock, but this often results in laxity and ptosis of the skin in the area.^7,8^

The thong lift (TL) procedure, presented in this paper, represents an innovative advancement in buttock aesthetics. Dr Constantino G. Mendieta, a well-regarded pioneer in the field of body contouring, has been a major contributor to the development and naming of the TL procedure; his work has been instrumental in advancing the TL approach.^9,10^ The procedure effectively targets inferomedial buttock ptosis and “boxy” lower contour deformities—deformities that fat transfer, buttock implants, and traditional buttock lifts often fail to fully correct.^11-13^ The TL is ideal for patients with isolated lower buttock issues, including those with lower pole ptosis from previous procedures. Patients, who have undergone extensive liposuction, are at the highest risk for developing lower pole ptosis of the buttock. The TL technique provides precise, anatomically targeted correction of the inferomedial buttock—an issue that cannot be resolved or corrected by simply adding volume to the buttock.

Unlike traditional buttock lifts or infragluteal thigh lifts—which are best for high/upper thigh laxity and lateral contour correction (inferolateral thigh laxity)—the TL targets the inferomedial buttocks to create a firm, lifted, and rounded look.^14-16^ By specifically addressing inferomedial ptosis, the TL procedure is able to correct L-shaped or boxy appearances of the buttock, often caused by factors like prior liposuction, weight loss, genetics, or aging.^17-20^

The TL procedure can be combined with other surgeries, such as the Brazilian butt lift (BBL) or buttock implants. Integrating the TL technique with buttock augmentation offers a distinct advantage, enhancing both volume and contour, leading to an improved aesthetic outcome for patients.^21-23^ This combined approach could improve patient satisfaction, especially for those who desire an overall improvement in the appearance and contour of their buttocks.

Because buttock aesthetics grow in popularity, particularly among patients who are seeking natural-appearing and anatomically specific results, the TL technique is a valuable tool that can be utilized by the plastic surgeon to enhance contour through the correction of lower buttock ptosis and smoothening of the L-shaped curve. In this paper, the authors will outline the TL procedure in depth and explore its ability to deliver excellent aesthetic outcomes, while also assessing its promise in being an effective addition to the existing repertoire of procedures for full buttock rejuvenation and contouring.

## METHODS

### Study Population

Patients underwent the TL procedure by 1 surgeon at a single private practice facility between January 2017 and April 2024. A retrospective chart review of all included patients was performed, and a survey was distributed to each patient to assess outcomes/results and patient satisfaction. Specifically, patient age, previous procedures, indication for TL, time to follow-up, postoperative complications, and TL type were recorded from patient charts. No patient was excluded from the study, and all patients were females. Informed consent was acquired from all patients. Photograph consent forms were obtained from patients if their photographs were included in this publication. No IRB approval was obtained for this study, and all ethical considerations align with the Declaration of Helsinki.

### Preoperative Evaluation/Markings

**Figure 1.**
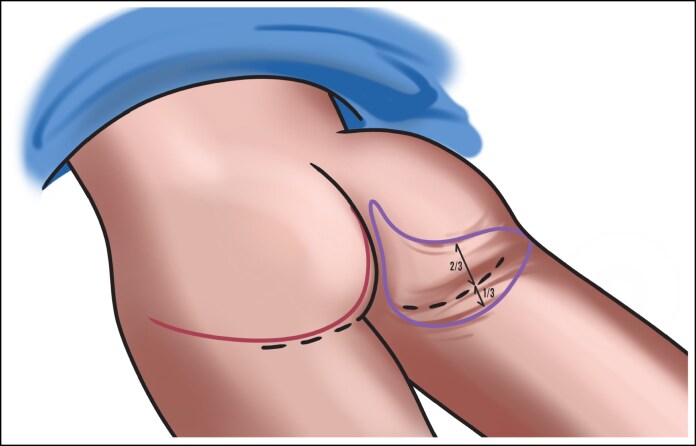
This illustration depicts the preoperative markings that should be made on the patient in the prone position when performing the thong lift procedure. On the left buttock, the traditional infragluteal thigh lift presurgical marking is shown. On the right buttock, the thong lift marking is shown. With respect to the thong lift markings, the patient should be examined from the posterior, with special attention paid to the degree of lower buttock ptosis and its orientation in the lateral or medial directions. The marking should be made using a pinch approximation technique, with 2/3 of the pinch above the infragluteal fold and 1/3 below. The markings should extend to the most lateral aspect of the infragluteal fold. The average width of the excision at its maximum is ∼8 cm. Notice the marked elevation/peak in the medial aspect of the superior marking—this must be achieved while marking in order to achieve ideal results. Artist Credit: Jim McConologue.

Each patient was examined, and the amount of lower buttock ptosis and degree to which the ptosis was evaluated. The amount of skin to be removed with an infragluteal horizontal excision was determined by pinching the skin, with ∼2/3 of the pinch located above the infragluteal fold and 1/3 of the pinch below the infragluteal fold. These marking lines extended to the most lateral aspect of the infragluteal fold, and, in the authors’ experience, the average width of the excision at its maximum length was ∼8 cm, on average. An illustration of the presurgical markings is showcased in Figure 1, and the entire preoperative marking process can be watched in detail in Video 1.

### Surgical Technique

**Figure 2.**
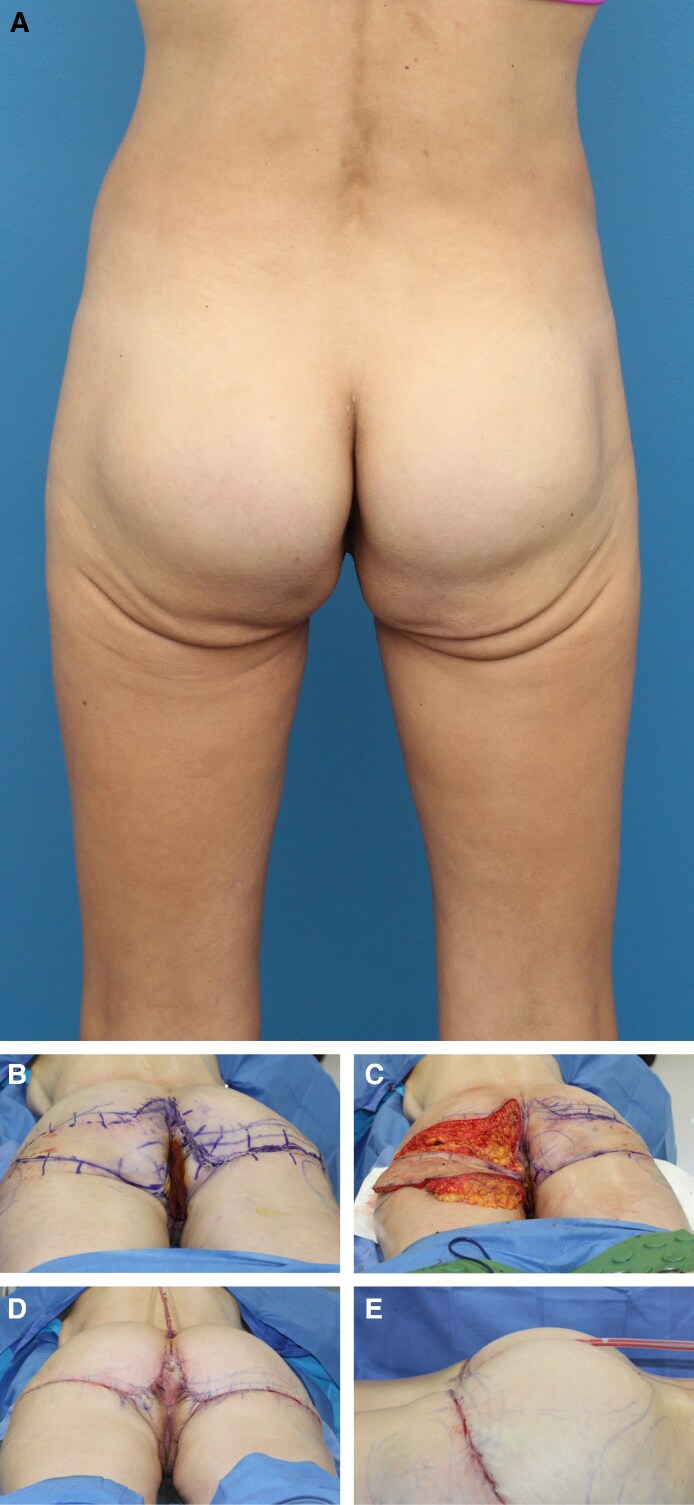
This patient is a 59-year-old female who presented with lower buttock ptosis, especially in the inferomedial region. The patient underwent the thong lift procedure, and her progression through the procedure and postoperative result is depicted in this 5-series figure. (A) A preoperative image. (B) The patient before the start of the operation with the R side tailor-tacked. No incisions have been made yet. (C) The patient midway through the procedure with incisions having been made on the left buttock first. (D) The patient immediately postoperatively on the operating table in the prone position. The natural and aesthetic final buttock contour can be appreciated. It is evident that there is minimal wrinkling medially, as the specified layered closure technique was utilized to minimize the wrinkling effect. However, the mild wrinkling that can be seen will subside significantly in the first 1 to 3 months after the procedure and will be completely resolve 4 to 6 months postoperatively. (E) The patient immediately postoperatively on the operating table in the prone position. The natural and aesthetic final buttock contour can be appreciated.

**Figure 3.**
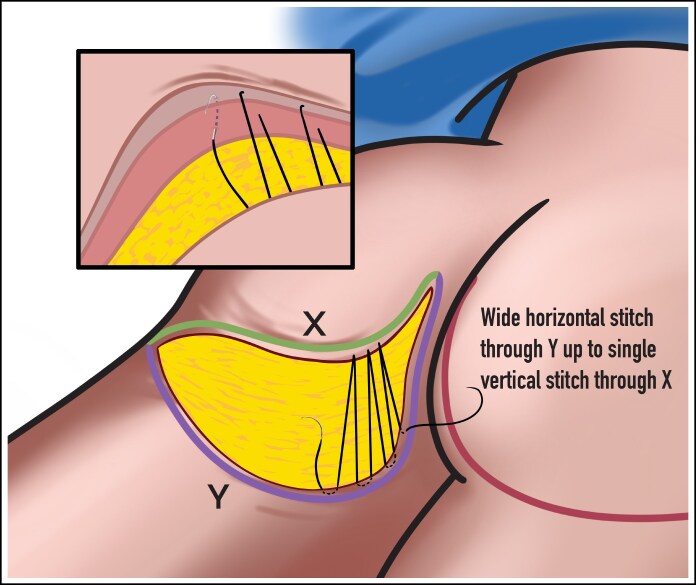
This illustration depicts the specialized closure technique that should be utilized when suturing the incision at the conclusion of the procedure. The incision must be closed in layers using a running 3-0 Vicryl suture in the subcutaneous tissues, a running 4-0 Monocryl suture in the dermis, and a running subcuticular 4-0 Monocryl suture. When each layer of the closure is performed, the superior flap bite must be vertical, whereas the inferior bite is longer in width horizontally. This specific technique is utilized to minimize the wrinkling that may be encountered during closure, because there is indeed a sizable discrepancy between the length of the shorter superior incision and the longer inferior incision. Artist credit: Jim McConologue.

**Figure 4.**
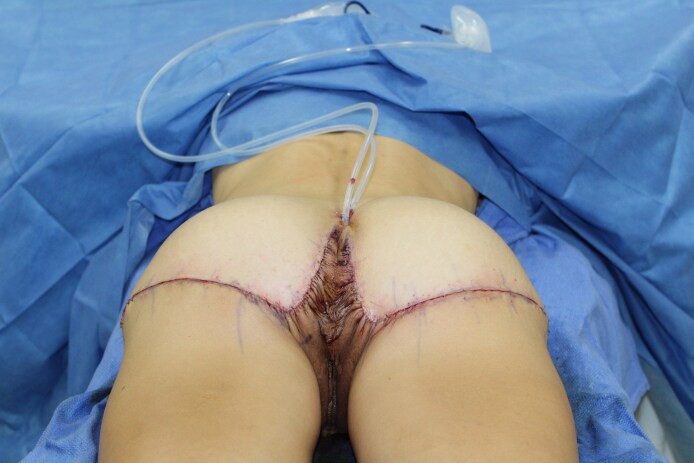
This patient is a 52-year-old female who presented with lower buttock ptosis, especially in the inferomedial region. The patient underwent the thong lift procedure and is shown here immediately postoperatively on the operating table in the prone position. The natural and aesthetic buttock contour can be appreciated. It is evident that there is minimal wrinkling medially as the specified layered closure technique was utilized to minimize the wrinkling effect. However, the mild wrinkling that can be seen will subside significantly 1 to 2 months after the procedure and will completely resolve 4 to 6 months postoperatively. Jackson–Pratt drains should be placed before closure and brought out through the inferior aspect of the sacrum. All incisions were dressed with SurgiSeal and Hypafix Tape in the earlier part of the study, and more recently with SYLKE dressing.

All procedures were performed in an accredited outpatient surgical facility under general anesthesia. Each patient was placed in a prone position, and the areas to be excised were injected with 1/2% lidocaine with epinephrine. A preoperative image is shown in Figure 2A. A tailor tack was performed with staples on both sides to assess how much tissue will need to be excised and to estimate and preview the results of the tissue excision before any actual incisions are made (Figure 2B). This step is crucial in the TL procedure because it helps to ensure symmetry. After making the appropriate markings and removing the staples from the tailor tack, the final incisions were made and the tissue specimens were removed (Figure 2C). A small portion of subcutaneous tissue was removed along the infragluteal fold, and oftentimes there is significant fibro-fatty tissue deposit in the inferior medial buttock that should be removed. The flaps were undermined 1 cm in each direction, and 7 mm Jackson–Pratt (JP) drains were placed and brought out through the inferior aspect of the sacrum. A drain is always placed in this procedure to manage postoperative fluid accumulation. When feasible, a 3-point suture is utilized to reduce dead space and shearing forces by securing the deep subcutaneous flap tissue to the underlying deep tissue. In cases where a 3-point stitch can be placed, the drain must be carefully manipulated out of the stitch path to allow for proper placement. However, there are anatomical regions where the tissue depth or density (excess tissue) hinders effective placement of a 3-point stitch. In these instances, although the drain must remain in place, the 3-point stitch may be omitted because of technical limitations. The incision was then closed in layers with a running 3-0 Vicryl (Ethicon, Inc., Somerville, NJ) suture in the subcutaneous tissues (3-point stitch), a running 4-0 Monocryl (Ethicon, Inc.) suture in the dermis, and a running subcuticular 4-0 Monocryl suture. With each layer of the closure, the superior flap bite should be vertical and the inferior bite should be long and horizontal (Figure 3). This technical modification is an important aspect of the procedure that serves to minimize the amount of wrinkling during the closure, because there is a sizable discrepancy between the length of the shorter superior incision and the longer inferior incision. All incisions were dressed with SurgiSeal (Adhezion Biomedical, LLC, Hudson, NC) and Hypafix Tape (Stockholm, Sweden) in the earlier part of the study and more recently with SYLKE (La Jolla, CA) dressing (Figures 2D, E, 4). For venous thromboembolism, prophylaxis compression stockings and Venodyne (Cardinal Health, Dublin, OH) sequential compression devices were utilized intraoperatively and postoperatively until the patient was able to ambulate. A full video outlining the surgical technique can be seen in Video 2.

### Postoperative Care and Infection Risk Mitigation

A mandatory follow-up visit was conducted on postoperative Day 1 for all patients. Because JP drains were placed intraoperatively, patients had their drains typically removed between postoperative Days 5 to 7, depending on output and clinical assessment. Patients were instructed to avoid buttock shearing and were given detailed postoperative instructions and guidance on appropriate movement and positioning. Specifically, they were advised to avoid any sliding or frictional movement between the buttocks while seated, such as scooting or shifting 1 gluteal region while the other remains fixed. Patients were advised to lift themselves vertically using armrests or support surfaces when adjusting their seated position and to stand by evenly shifting their weight through both legs. Additionally, lying and sitting in symmetrical positions were strongly encouraged, and patients were advised to minimize lateral torque or twisting of the gluteal region. Otherwise, patients were permitted to ambulate freely and sleep in the supine position without restriction.

Preoperative antibiotic prophylaxis consisted of 1 g intravenous Vancomycin (Hospira, Lake Forest, IL) after induction and Tobramycin (Hospira; dosing based on weight gram/kilogram and normal dosing at induction). Betadine (Purdue Products L.P., Stamford, CT) was applied intraoperatively, and betadine-soaked gauze was secured to the perianal region using staples. Postoperatively, all patients received 500 mg oral Clindamycin (Pfizer Inc., New York, NY) 3 times daily for 2 days. With regard to wound dressing, SurgiSeal was applied initially postoperatively and was removed on postoperative Day 5. Patients were then instructed to apply Hypafix Tape (BSN Medical GmbH, Hamburg, Germany) for an additional 2 weeks. Patients were also instructed to maintain good hygiene and clean the area carefully to further minimize the risk of infection. Utilization of a postoperative garment was determined on a case-by-case basis for each patient and was based on which procedures were performed. For patients who underwent additional liposuction, a standard postoperative compression garment was recommended to be worn at all times for 2 weeks and then only at night for 1 week. In cases where no liposuction was performed, a gauze dressing secured with mesh brief panties was utilized. No additional compression garments were utilized in those instances.

### Patient-Reported Outcome Measures

All patients were contacted postoperatively and asked to complete a comprehensive questionnaire regarding their procedure, outcomes, and overall satisfaction. The survey utilized in this study was an expert-designed form designed to measure patient-reported satisfaction. Patients were contacted through email or phone following their surgery, typically 1 year postoperatively, to ensure an appropriate recovery period before patient outcomes were obtained. Patients were able to rate various parameters about their outcome using a Likert Scale (1-10). A score of 10 on the scale represents the best possible patient-reported outcome for a specific measure or parameter, whereas a score of 1 represents the worst possible outcome for that measure or parameter. All patients rated their results for each of the following parameters: (1) the improvement in lower buttock ptosis, (2) the improvement in contour of the lower buttocks, (3) visibility of the scar at the incision site, and (4) quality of the scar at the incision site. A basic set of statistical analyses was performed using the results from the patient survey. The mean, standard deviation (SD), and range of all patient scores from the Likert Scale were calculated.

## RESULTS

A total of 26 patients underwent the TL surgical procedure. All patients were operated on using the exact same technique as described above. The mean age was 48.46 years (range, 27-69 years), and the mean follow-up period was 48.7 months (range, 12-110 months; Table 1). The average BMI was 21.52 kg/m^2^. Among 26 patients, 26.9% of patients (7/26) had a previous BBL surgery, 11.5% of patients (3/26) had a previous thigh lift, and 50% of patients (13/26) had previous liposuction performed. Approximately 11.5% of (3/26) patients had undergone significant weight loss from GLP-1 medication. In addition, 46.2% of patients (12/26) underwent combination procedures where TL was combined with either implants and/or a BBL. Two patients underwent TL with concurrent BBL and implants. Seven patients underwent TL with concurrent BBL only, whereas 3 patients underwent TL with concurrent implants only. About 7.7% (2/26) of patients had hypertension, and about 3.8% of patients had diabetes (1/26). Approximately 11.5% (3/26) patients had a history of smoking. A comprehensive table outlining all relevant patient demographics and characteristics for each patient can be seen in Supplemental Table 1. The average specimen dimension was 8 × 32 cm, which was the typical volume of excess tissue that needed to be excised in order to adequately address the lower buttock ptosis and achieve the desired contour enhancement the patients desired.

**Table 1.** Summary of Patient Demographics

Characteristic	Value
Average age, years (range)	48.5 (27-69)
Gender, *n* (%)	
Female	26 (100)
Male	0 (0)
Average BMI (kg/m^2^)	21.5 (16.5-27.6)
Average follow-up (months)	48.7 (12-110)
Patients with smoking Hx, *n* (%)	3 (11.5)
Complications, *n* (%)	
Major	0 (0)
Minor	4 (15.4)
Seroma	2 (50)
Scar revision	1 (25)
Wound dehiscence^a^	1 (25)
TL type, *n* (%)	
TL alone (no BBL or implants)	14 (53.8)
TL with BBL	7 (26.9)
TL with implants	3 (11.5)
TL with BBL and implants	2 (7.7)
Indication for procedure, *n* (%)	
Cosmetic/appearance of buttock	23 (88.5)
Post-GLP-1 weight loss	3 (11.5)
Comorbidities, *n* (%)	
Hypertension	2 (7.7)
Diabetes mellitus	1 (3.8)
Previous procedures, *n* (%)	
Breast augmentation	19 (73.1)
Liposuction	13 (50)
BBL	7 (26.9)
Mastopexy	7 (26.9)
Abdominoplasty	6 (23.1)
Facelift	5 (19.2)
Thigh lift	3 (11.5)

BBL, Brazilian butt lift; TL, thong lift.

^a^<1 cm wound opening.

**Figure 5.**
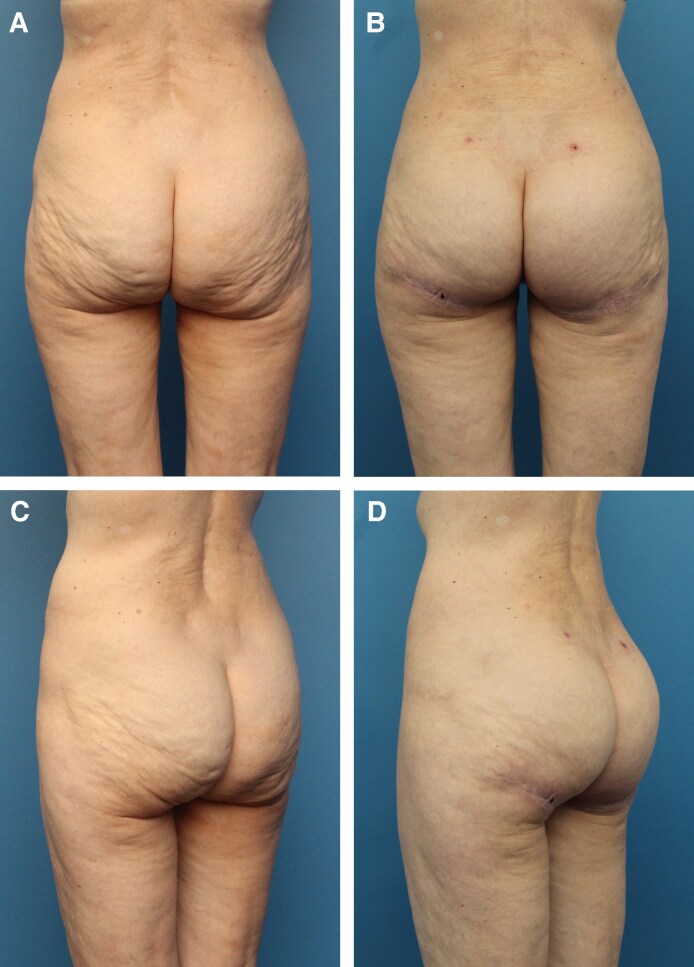
A 59-year-old female with lower buttock ptosis, particularly in the inferomedial region of the buttock. (A, C) Preoperative; (B, D) at 6 months, following the thong lift procedure, liposuction of the hips, and fat transfer to the buttocks (Brazilian butt lift). A total of 300 cc of fat was harvested from each hip and 100 cc from each outer thigh. Ultimately, a total of 140 cc of fat was transferred to the upper pole of the buttocks. Complete resolution of the inferomedial buttock ptosis can be seen, offering a smooth and natural contour. Note: the 2 incisions visible in the lower back are the access sites utilized for liposuction.

**Figure 6.**
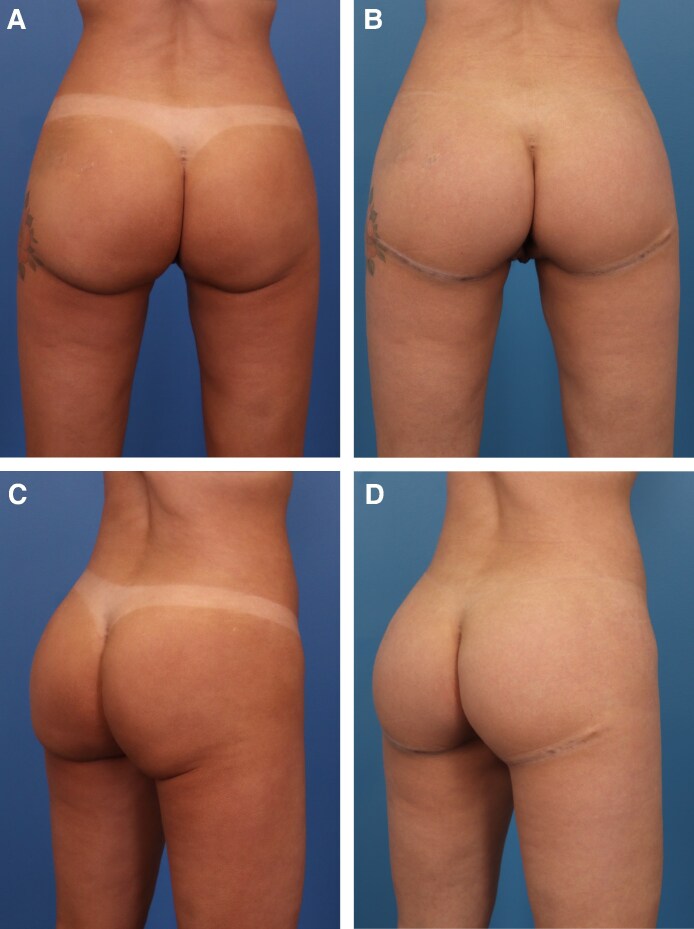
A 41-year-old female with lower buttock ptosis, particularly in the inferomedial region of the buttock. This patient had previously undergone high-definition liposuction of the buttocks, a procedure commonly found in the history of patients presenting with this type of buttock contour. (A, C) Preoperative; (B, D) at 1 year following thong lift procedure. Complete resolution of the inferomedial buttock ptosis can be seen, offering a smooth and natural contour.

Before and after results of 2 different patients can be seen in Figures 5 and 6. Both patients presented with inferomedial buttock ptosis with an L-shaped and boxy deformity. They underwent the TL procedure, resulting in full correction of the lower buttock ptosis and a natural and aesthetic buttock contour. In Figures 5 and 6, 1 year- and 6-month postoperative results of patients are shown, respectively.

A retrospective review of all patients was performed, and a questionnaire was sent to all patients. According to the patient-reported outcomes rating, the improvement in lower buttock ptosis received an average rating of 9.4/10 (SD: 0.99; range, 6-10; Table 2). The contour of the lower buttocks was also rated at 9.4/10 (SD: 1.02; range, 6-10). The visibility of the scar at the incision site was rated 7.4/10 (SD: 2.17; range, 3-10), whereas the quality of the scar at the incision site received a rating of 7.8/10 (SD: 1.39; range, 5-10). These data, as well as the median values, are summarized in Table 2.

**Table 2.** Patient-Reported Outcome Measure/Satisfaction Ratings

	Improvement in lower buttock ptosis	Contour of lower buttocks	Visibility of the scar at the incision site	Quality of the scar at the incision site
Average patient rating (SD) (Likert Scale 1-10)^a^	9.4 (0.99)	9.4 (1.02)	7.4 (2.17)	7.8 (1.39)
Median	10	10	8	8
Range	6-10	6-10	3-10	5-10

SD, standard deviation.

^a^In the Likert Scale questionnaire administered to patients, a score of 10 represents the most optimal patient-reported outcome, whereas a score of 1 indicates the least optimal patient outcome.

There were no major complications (0/26) observed. However, 4/26 (15.4%) patients did experience minor complications. Minor wound dehiscence (<1 cm wound openings) occurred in 1/26 (3.8%) cases but resolved with local wound care, including debridement and topical ointment-based dressing changes ∼2 weeks postoperatively. Additionally, 2/26 (7.7%) patients developed seromas, both of which required 2 aspirations each before fully resolving. Lastly, 1/26 (3.8%) patients required a scar revision surgery, where a localized re-excision was performed because of stretching of the skin and residual redundancy of the skin.

## DISCUSSION

The results were generally positive in all 26 patients who underwent the TL procedure, and the majority of patients were very pleased with the contour and scar appearance. Scores for patient satisfaction indicate that the TL successfully meets aesthetic demands, including correcting ptosis and obtaining a symmetrical, rounded buttock.

Surgeons perform the TL introduce cuts in areas where scarring is less apparent, which leads to aesthetically pleasing treatments with minimal visible scarring.^24-26^ Additionally, controlled excision and precise closure of the TL prevent the development of scar complications and optimize long-term patient outcomes.^27,28^

### Technical Considerations

A key technical feature of the TL is the difference in incision lengths between the upper and lower half, reflecting the anatomy of the buttock and allowing for accurate control of the lower pole shape. The superior incision is relatively narrow and has a medial upside-down parabolic indentation at the upper edge, whereas the inferior one is longer and horizontal in shape, allowing for trimming away the excess tissue and shaping the lower pole (Figure 1). This is achieved by closing the shorter incision with vertical sutures, whereas the longer, more inferior incision is closed with horizontal sutures, a special technique further discussed later in the paper. This design ensures the smaller upper side is securely attached to the larger, lower side, evenly distributing tension around the wound and avoiding puckering or other defects in the suture line.

The vertical sutures on the shorter end and horizontal sutures on the longer end are an optimized closure strategy for the incision, reducing tension while aiding the overcorrection goal (Figure 3). This method is utilized to mitigate the potential wrinkling that could occur postoperatively because of the significant disparity in the lengths of the upper and lower incision. Furthermore, precise intraoperative lines—generally 23 cm above the infragluteal fold and 13 cm below—allow for a precise excision that provides the ideal combination of lift and natural buttock profile. The average excision width in the authors’ experience is ∼8 cm. At the end of the procedure, as noted in Video 2, one may notice a “splaying” of the buttocks, which describes the stretched-out visual appearance of the buttock when the patient is lying in the prone position. This may look abnormal at first but has not proved to be problematic in our series, as there have been no complications because of this. Once the patient stands up, the “splaying” of the buttocks resolves.

### Thong Lift vs Standard Infragluteal Thigh Lift

The most significant difference between the TL and traditional infragluteal thigh lift is the fact that it targets not the thigh, but the buttock itself. The classic infragluteal thigh lift is useful for medial thigh laxity, but it cannot adequately address inferomedial age-related sagging or the boxy shape of the lower buttock.^29-31^ TL's singular focus thus complements the range of body contouring procedures by correcting the particular shape of the lower buttock that thigh lifts cannot cover.^30^

### Postoperative Wrinkling and Resolution

Wrinkling of the lower buttock skin near the incision is a common postoperative occurrence in the early stages of healing, typically improving significantly within the first 1 to 2 months after surgery. For example, 1 patient in this study experienced severe wrinkling 2 weeks postsurgery which gradually subsided by the 1- and 3-month time points. The wrinkling was fully resolved by the 6-month follow-up visit, leaving a visible scar with no wrinkling. In most patients, the wrinkling should be expected to completely subside anywhere between 4 and 6 months postoperatively. Such distribution of tension on the wound is sufficient in that it heals irrespective of the differences in length of the incisions made, naturally allowing the early creases to gradually remodel over time. As such, the development of wrinkling in the early stages of healing is an expected process and should be explained to the patients as such. It could take up to a few months for the wrinkling and folds to resolve after surgery. This clarification is essential for ensuring high postoperative satisfaction, because it reassures patients that mild wrinkling will not affect the long-term aesthetic outcome. Instead, it is a temporary phase in the skin's natural adaptation process after surgery.

### Blending Thong Lift With Other Techniques to Achieve Better Results

The TL can be complemented with other cosmetic surgeries, including the BBL and implants, to optimize inferomedial buttock contouring for patients seeking additional augmentation. This is especially helpful for patients presenting with both lower buttock ptosis and generalized volume loss. Fat grafting or implants combined with the TL create a better buttock contour, restoring shape and volume simultaneously.^32,33^ These procedures allow surgeons to establish an anatomically accurate profile, achieving the desired aesthetic outcome of a naturally firm appearance.

These combined procedures are often helpful in patients with notable anatomical variations arising from significant weight loss or liposuction. Several patients who underwent the TL procedure had concurrent BBLs and implants to correct both lower buttock ptosis and restore volume. These patients achieved a natural and full-appearing buttock and were overwhelmingly satisfied with their results. This hybrid technique corrects the L-shaped defect while restoring a youthful profile to the lower pole, resulting in optimal patient satisfaction.

In all combined procedures, the TL is performed last. For patients undergoing buttock augmentation with implants, the access incision typically achieved through a paramedian approach is incorporated into the TL excision. This allows for a single, concealed incision site and improved aesthetic outcomes. If fat grafting is performed, it is completed after implant placement but before the closure of the TL incision.

### Limitations

There are several limitations to this study. The greatest limitation is the small sample size of 26 patients, which limits the greater generalizability of the findings from this study. Additionally, this study is a single-center, single-surgeon study. This may have introduced bias into the study with regard to patient selection, surgical technique, and outcome assessment. A multicenter study including multiple surgeons would be useful to validate the results of this study. Lastly, utilizing more precise and targeted measurement tools for patient-reported outcome measures (PROMs) in buttock procedures, such as Body-Q—which, like Breast-Q, is designed for body contouring—would have enabled us to perform a more robust analysis of PROMs and overall patient satisfaction.

## CONCLUSIONS

The TL technique can be utilized to correct lower buttock ptosis and contour irregularities that are not effectively corrected by conventional techniques, such as fat transfer, implants, or traditional buttock lifts. It anatomically targets the inferomedial lower buttock and is particularly suited for patients with L-shaped or boxy deformities. The TL can also be combined with procedures such as the BBL and buttock implantation, further expanding its applicability for structural correction and volume enhancement.

## Supplemental Material

This article contains supplemental material located online at https://doi.org/10.1093/asjof/ojaf100.

## Supplementary Material

ojaf100_Supplementary_Data

## References

[1] Aesthetic Plastic Surgery National Databank Statistics 2023. Aesthet Surg J. 2024;44:1–25. doi: 10.1093/asj/sjae188 39283306

[2] Pascal JF. Buttock lifting: the golden rules. Clin Plast Surg. 2019;46:61–70. doi: 10.1016/j.cps.2018.08.008 30447829

[3] Mowlavi A, Berri M, Talle A, Talle M. Objectifying high-definition Brazilian buttock lift results using the buttock assessment tool. Plast Reconstr Surg. 2021;148:727e–734e. doi: 10.1097/PRS.0000000000008479 34705775

[4] Mowlavi A, Sin Z, Sahami C, Mantecon G, Mirzania H. Optimizing Brazilian buttock lift results using the BBL assessment tool. Aesthetic Plast Surg. 2023;47:666–681. doi: 10.1007/s00266-022-03120-1 36214875

[5] Lee EI, Roberts TL, Bruner TW. Ethnic considerations in buttock aesthetics. Semin Plast Surg. 2009;23:232–243. doi: 10.1055/s-0029-1224803 20676318 PMC2884922

[6] Centeno RF, Sood A, Young VL. Clinical anatomy in aesthetic gluteal contouring. Clin Plast Surg. 2018;45:145–157. doi: 10.1016/j.cps.2017.12.010 29519484

[7] Gonzalez R. Treating the banana fold with the dermotuberal anchorage technique: case report. Aesthetic Plast Surg. 2005;29:300–303. doi: 10.1007/s00266-004-0133-2 15940397

[8] Alves HRN, Nicolas G. Gluteoplasty with lumbar gluteal flap associated with liposuction and fat grafting: a safe technique for massive weight loss patients. Aesthet Surg J. 2024;44:404–411. doi: 10.1093/asj/sjad339 37879116

[9] The art of gluteal sculpting. Plast Reconstr Surg. 2012;130:736. doi: 10.1097/PRS.0b013e318269c48b

[10] Writer AW. An Alternative to the Brazilian Butt Lift: what is the Miami Thong Lift? American Society of Plastic Surgeons; 2024. Accessed May 11, 2025. https://www.plasticsurgery.org/news/articles/an-alternative-to-the-brazilian-butt-lift-what-is-the-miami-thong-lift

[11] Gonzalez R. Etiology, definition, and classification of gluteal ptosis. Aesthetic Plast Surg. 2006;30:320–326. doi: 10.1007/s00266-005-0051-y 16733776

[12] Coban YK, Uzel M, Celik M. Correction of buttock ptosis with anchoring deepithelialized skin flaps. Aesthetic Plast Surg. 2004;28:116–119. doi: 10.1007/s00266-004-3109-3 15170248

[13] Yang M, Li J, Dong W, et al Lower gluteal liposuction combined with upper gluteal and infragluteal region fat grafting: a novel concept to improve gluteal ptosis. Aesthet Surg J. 2024;44:NP329–NP336. doi: 10.1093/asj/sjae027 38324894

[14] Hamra ST, Small KH. Cosmetic body lift. Plast Reconstr Surg. 2016;137:453–461. doi: 10.1097/01.prs.0000475757.56086.ab 26818279

[15] Capella JF. Body lift. Clin Plast Surg. 2008;35:27–51; discussion 93. doi: 10.1016/j.cps.2007.08.001 18061797

[16] Musmarra I, Aguilar P, Struk S, Couteau C, Tresallet C, Quilichini J. Vertical body lift: surgical technique and comparison with the inferior body lift technique. Plast Reconstr Surg. 2023;152:507e–517e. doi: 10.1097/PRS.0000000000010291 36780353

[17] Boswell CB. Body contouring following massive weight loss. Mo Med. 2010;107:189–194. PMID: 20629287. PMCID: PMC6188338. 20629287 PMC6188338

[18] Dixit VV, Wagh MS. Unfavourable outcomes of liposuction and their management. Indian J Plast Surg Off Publ Assoc Plast Surg India. 2013;46:377–392. doi: 10.4103/0970-0358.118617 PMC3901919 24501474

[19] Shrivastava P, Aggarwal A, Khazanchi RK. Body contouring surgery in a massive weight loss patient: an overview. Indian J Plast Surg Off Publ Assoc Plast Surg India. 2008;41:S114–S129. PMCID: PMC2825131. PMID: 20174535. PMC2825131 20174535

[20] Mahgoub MA, Zeina AM, Bahaa El-Din AM, El-Sabbagh AH, Bassetto F, Vindigni V. Gluteal region reshaping of massive weight loss patients—a decision-making strategy. Arch Plast Surg. 2022;49:289–295. doi: 10.1055/s-0042-1748640 35832159 PMC9142265

[21] Cansancao AL, Condé-Green A, Gouvea Rosique R, Junqueira Rosique M, Cervantes A. Brazilian butt lift” performed by board-certified Brazilian plastic surgeons: reports of an expert opinion survey. Plast Reconstr Surg. 2019;144:601–609. doi: 10.1097/PRS.0000000000006020 31461012

[22] Rios L, Gupta V. Improvement in Brazilian butt lift (BBL) safety with the current recommendations from ASERF, ASAPS, and ISAPS. Aesthet Surg J. 2020;40:864–870. doi: 10.1093/asj/sjaa098 32306045

[23] Güzey S, Ergan Şahin A. Brazilian butt lift: an experience over 3000 patients. Aesthetic Plast Surg. 2024;48:2677–2693. doi: 10.1007/s00266-024-03965-8 38580866

[24] Mejia JA. Gluteal reduction: a new technique with tightening, lifting, and reshaping effects on the buttocks. Aesthetic Plast Surg. 2012;36:550–556. doi: 10.1007/s00266-011-9863-0 22286866

[25] Khansa I, Harrison B, Janis JE. Evidence-based scar management: how to improve results with technique and technology. Plast Reconstr Surg. 2016;138:165S–178S. doi: 10.1097/PRS.0000000000002647 27556757

[26] Garg S, Dahiya N, Gupta S. Surgical scar revision: an overview. J Cutan Aesthetic Surg. 2014;7:3–13. doi: 10.4103/0974-2077.129959 PMC3996787 24761092

[27] Azmat CE, Council M. Wound closure techniques. In: StatPearls. StatPearls Publishing; 2025. Accessed January 17, 2025. http://www.ncbi.nlm.nih.gov/books/NBK470598/29262163

[28] Ogawa R. Ideal wound closure methods for minimizing scarring after surgery. In: Téot L, Mustoe TA, Middelkoop E, Gauglitz GG, ed. Textbook on Scar Management: State of the Art Management and Emerging Technologies. Springer; 2020:186–187. http://www.ncbi.nlm.nih.gov/books/NBK586099/. 36351136

[29] Kolker AR, Xipoleas GD. The circumferential thigh lift and vertical extension circumferential thigh lift: maximizing aesthetics and safety in lower extremity contouring. Ann Plast Surg. 2011;66:452–456. doi: 10.1097/SAP.0b013e3182145682 21451373

[30] Bell D, Winters R. Thighplasty. In: StatPearls. StatPearls Publishing; 2025. Accessed January 17, 2025. http://www.ncbi.nlm.nih.gov/books/NBK594236/37603627

[31] Kirwan L. Anchor thighplasty. Aesthet Surg J. 2004;24:61–64. doi: 10.1016/j.asj.2003.10.003 19336138

[32] Gonzalez R. Buttocks lifting: the dermo-tuberal anchorage technique. Aesthet Surg J. 2005;25:15–23. doi: 10.1016/j.asj.2004.11.003 19338782

[33] Hoenig JF, Swetje K. Age-related ptosis of the buttock: an anthropometric gender-specific analysis. Aesthetic Plast Surg. 2013;37:1090–1099. doi: 10.1007/s00266-013-0205-2 24114293

