# Supplementary material for: Technical Aspects of Addressing Lower Buttock Ptosis and Contouring: The Thong Lift Technique
Source: Aesthet Surg J Open Forum. 2025 Aug 8;7:ojaf100. doi: 10.1093/asjof/ojaf100 (PMC12548050; doi:10.1093/asjof/ojaf100)
Supplement: ojaf100_Supplementary_Data [file ojaf100_Supplementary_Data.docx]

**Supplemental Table 1. Patient Demographics**

| **PATIENT NUMBER** | **PATIENT AGE (YEARS)** | **PREVIOUS PROCEDURES** | **REASON FOR PROCEDURE** | **FOLLOW-UP (MONTHS)** | **POST OPERATIVE COMPLICATIONS** | **THONG LIFT TYPE** | **SMOKER** | **BMI (kg/m^2^)** | **COMORBIDITIES** |
| --- | --- | --- | --- | --- | --- | --- | --- | --- | --- |
| 1 | 60 | Breast Augmentation, Lower Facelift, Brow Lift, Lip Implants | Weight loss from GLP-1's (70 lbs.) with a desire to improve her buttock appearance and contour | 12 | Major Complications: None   Minor Complications: None | Thong Lift with BBL and Implants | Yes | 20.2 | None |
| **2** | **45** | **Liposuction, Body lift, BBL, Thigh Lift** | **Weight loss from GLP-1's, Desire for improvement in the appearance of buttocks.** | **12** | **Major Complications: None   Minor Complications: None** | **Thong Lift with Implants** | **No** | **22.1** | **Diabetes** |
| 3 | 40 | Endoscopic Browlift, Chin Augmentation, Fat Transfer -Face, Rhinoplasty, Upper Blepharoplasty, Breast Augmentation, Mastopexy, Liposuction, BBL, Upper Lip Lift, | Desire for improvement in the appearance of lower buttock. | 14 | Major Complications: None   Minor Complications: None | Thong Lift (No Implants or BBL) | No | 18.6 | None |
| **4** | **35** | **Breast Augmentation, Thigh Lift, Abdominoplasty, Rhinoplasty, Septoplasty** | **Desire for improvement in the appearance of buttock.** | **17** | **Major Complications: None   Minor Complications: None** | **Thong Lift with BBL** | **No** | **25.7** | **None** |
| 5 | 41 | Breast Augmentation, Left Mastopexy, Explantation, Fat Transfer | Desire for improvement in appearance of lower buttock. | 17 | Major Complications: None   Minor Complications: Seroma | Thong Lift with BBL | No | 21.1 | None |
| **6** | **38** | **Breast Augmentation, Re-Augmentation, Re-Augmentation, Mini Abdominoplasty, Liposuction, Capsulotomy** | **Desire for improvement in appearance of lower buttock.** | **18** | **Major Complications: None   Minor Complications: None** | **Thong Lift (No Implants or BBL)** | **No** | **18.3** | **None** |
| 7 | 35 | Upper Blepharoplasty, Breast Augmentation , Mastopexy, Liposuction - outer thighs | Desire for improvement in the appearance of buttock. | 22 | Major Complications: None   Minor Complications: None | Thong Lift (No Implants or BBL) | No | 21.3 | None |
| **8** | **27** | **Breast Augmentation** | **Desire for improvement in the appearance of lower buttock redundancy** | **21** | **Major Complications: None   Minor Complications: None** | **Thong Lift (No Implants or BBL)** | **No** | **24.9** | **None** |
| 9 | 57 | Implant Exchange, Revision Umbilicus, Breast Augmentation, Mastopexy | Weight loss from GLP-1's ) with a desire to improve her buttock appearance and contour | 27 | Major Complications: None   Minor Complications: Scar Revision | Thong Lift with Implants | No | 22.2 | None |
| **10** | **59** | **Breast Augmentation, Liposuction, Blepharoplasty** | **Desire to improve appearance of buttock** | **33** | **Major Complications: None   Minor Complications: None** | **Thong Lift with BBL** | **No** | **20.3** | **None** |
| 11 | 53 | Breast Reduction, Breast Augmentation, Mini Abdominoplasty, Butt Lift and Breast Lift | Desire to improve appearance of buttock | 39 | Major Complications: None   Minor Complications: None | Thong Lift with BBL | No | 23.2 | None |
| **12** | **42** | **Rhinoplasty, Facelift** | **Desire to improve appearance of buttock** | **41** | **Major Complications: None   Minor Complications: None** | **Thong Lift with BBL** | **Yes** | **27.6** | **None** |
| 13 | 53 | Breast Augmentation, Mastopexy, Liposuction | Desire to improve appearance of buttock | 56 | Major Complications: None   Minor Complications: None | Thong Lift with Implants | No | 21.3 | None |
| **14** | **51** | **Abdominoplasty, Breast Augmentation, Mastopexy** | **Desire to improve appearance of buttock** | **46** | **Major Complications: None   Minor Complications: None** | **Thong Lift with Implants and BBL** | **Yes** | **18.4** | **None** |
| 15 | 60 | Thigh Lift, Beltline Lipectomy, Lip Lift, Knee Lift | Desire to improve appearance of buttock | 50 | Major Complications: None   Minor Complications: Seroma | Thong Lift (No Implants or BBL) | No | 17.4 | None |
| **16** | **42** | **Belt Lipectomy, Fat Transfer Liposuction** | **Desire to improve appearance of buttock** | **51** | **Major Complications: None   Minor Complications: None** | **Thong Lift (No Implants or BBL)** | **No** | **23.3** | **None** |
| 17 | 53 | Buttock Implants, Liposuction, Abdominoplasty, Breast Augmentation, Implant Exchange | Desire to Improve appearance of buttock | 53 | Major Complications: None   Minor Complications: None | Thong Lift(No Implants or BBL) | No | 16.5 | None |
| **18** | **64** | **Brachioplasty, Lip Lift, Facelift, Browlift, Earlobe Reduction** | **Desire to improve appearance of buttock** | **62** | **Major Complications: None   Minor Complications: None** | **Thong Lift (No Implants or BBL)** | **No** | **20** | **High Blood Pressure** |
| 19 | 56 | Liposuction, BBL | Desire to improve appearance of buttock | 68 | Major Complications: None   Minor Complications: None | Thong Lift (No Implants or BBL) | No | 21.3 | None |
| **20** | **48** | **Liposuction, BBL, Thigh lift, Buttock Implants, Reverse Tummy Tuck** | **Desire to improve appearance of buttock** | **70** | **Major Complications: None   Minor Complications: None** | **Thong Lift (No Implants or BBL)** | **No** | **18.3** | **None** |
| 21 | 62 | Facelift, Abdominoplasty, Liposuction | Desire to improve appearance of buttock | 78 | Major Complications: None   Minor Complications: None | Thong Lift (No Implants or BBL) | No | 23 | None |
| **22** | **48** | **Breast Augmentation** | **Desire to improve appearance of buttock** | **85** | **Major Complications: None   Minor Complications: Wound Dehiscence** | **Thong Lift with BBL** | **No** | **21.6** | **None** |
| 23 | 32 | Breast Augmentation | Desire to improve appearance of buttock | 87 | Major Complications: None   Minor Complications: None | Thong Lift (No Implants or BBL) | No | 23 | None |
| **24** | **48** | **Abdominoplasty, Body lift** | **Desire to improve appearance of buttock** | **88** | **Major Complications: None   Minor Complications: None** | **Thong Lift (No Implants or BBL)** | **No** | **27** | **None** |
| 25 | 69 | Facelift, Breast Reduction, Liposuction | Desire to improve appearance of buttock | 89 | Major Complications: None   Minor Complications: None | Thong Lift (No Implants or BBL) | No | 18.6 | High Blood Pressure |
| **26** | **42** | **Mastopexy, Liposuction** | **Desire to improve appearance of buttock** | **110** | **Major Complications: None   Minor Complications: None** | **Thong Lift with BBL** | **No** | **24.2** | **None** |
| **Average:** | **48.5** | **N/A** | **N/A** | **48.7** | Major Complications: None   Minor Complications: None | **N/A** | **N/A** | **21.52** | **N/A** |
